# A calcium-mobilizing biostimulant provides tipburn control comparable to vertical airflow fans in greenhouse hydroponic lettuce ‘Rex’

**DOI:** 10.3389/fpls.2025.1701667

**Published:** 2025-11-28

**Authors:** Moein Moosavi-Nezhad, Qingwu Meng

**Affiliations:** 1 Department of Plant and Soil Sciences, University of Delaware, Newark, DE, United States; 2 Department of Horticultural Science, North Carolina State University, Raleigh, NC, United States

**Keywords:** calcium deficiency, calcium mobility, controlled environment agriculture, deep water culture, nutrient deficiency

## Abstract

Lettuce (*Lactuca sativa*) tipburn is a calcium-related physiological disorder affecting young, enclosed leaves, reducing marketability. Vertical airflow fans (VAFs) effectively control tipburn in greenhouses but are limited by installation complexity, shading, and energy use. A chemical biostimulant can be added to hydroponic nutrient solution to enhance calcium mobility and reduce tipburn, but its efficacy relative to VAFs is unknown. We evaluated three biostimulant concentrations (0, 0.25, and 0.5 mL·L^−1^) with and without VAFs (≈1 m·s^−1^) in a split-plot randomized complete block design using greenhouse deep-water-culture hydroponics in summer. Lettuce ‘Rex’ plants were assessed 14, 21, and 28 days after transplant (DAT). Tipburn severity rating (on a scale from 0 = none to 5 = severe) and the percentage of leaves affected increased over time in the control (NoVAFs, no biostimulant), reaching 5.0 and 39%, respectively, at 28 DAT. Application of the calcium-mobilizing biostimulant at 0.5 mL·L^−1^ reduced tipburn by 94%–96% and 71%–75% at 21 and 28 DAT, respectively, under NoVAFs, comparable to the elimination or minimization of tipburn by VAFs. Shoot fresh and dry mass were transiently reduced by 16%–32% at 14–21 DAT under the highest biostimulant concentration, but by 28 DAT, biomass was comparable to the control across treatments, and minor reductions in tissue moisture content under VAFs explained fresh-mass differences. Inner-leaf calcium concentrations increased with biostimulant application under NoVAFs, correlating inversely with tipburn severity, whereas VAFs promoted calcium delivery independently of the biostimulant. These results demonstrate that the calcium‐mobilizing chemical biostimulant results in comparable tipburn mitigation in hydroponic lettuce to VAFs under summer greenhouse conditions, with no yield penalty at final harvest, and can be used where VAFs are impractical.

## Introduction

Lettuce (*Lactuca sativa*) tipburn is a physiological disorder characterized by the browning, necrosis, and curling of leaf tips and margins and primarily affects young leaves in the enclosed center of the lettuce head. Its underlying cause is localized calcium deficiency in the actively growing region due to a relative imbalance of calcium demand (driven by the plant growth rate) exceeding calcium supply (Barta and Tibbitts, 2000; Saure, 1998). Calcium plays a critical role in maintaining cell wall structure and membrane stability, particularly in actively growing tissues. Its uptake and movement within the plant are largely dependent on transpiration, which drives water movement through the plant in a process known as mass flow (Clarkson, 1984). Calcium is absorbed by roots and translocated to shoots and leaves via the xylem. As a generally immobile nutrient, once deposited, calcium cannot easily move from older to newer tissues. Therefore, calcium deficiency affects newer leaves, which are highly dependent on sufficient transpiration and thus mass flow to maintain adequate calcium supply (Barta and Tibbitts, 2000).

Despite sufficient calcium availability in the root zone, tipburn still occurs under transpiration-limiting environmental conditions (e.g., high relative humidity and inadequate air movement), which inhibit calcium uptake and supply, and is exacerbated by growth-promoting environmental conditions (e.g., high light and temperature), which increase calcium demand (Frantz et al., 2004). Tipburn severity in hydroponic lettuce is influenced by genotype and associated with altered leaf nutrient composition, notably calcium and allied ions (Birlanga et al., 2021). Reporting cultivar details is therefore critical when interpreting treatment effects on tipburn. Although some lettuce cultivars are more resistant to tipburn than others, even the least susceptible cultivars from trials can develop tipburn under the above conducive conditions (Ertle and Kubota, 2023a). Tipburn significantly reduces the visual quality and sellable yield of lettuce, thereby a constant economic threat to profitability in controlled-environment agriculture (Uno et al., 2016).

A variety of strategies have been devised to mitigate lettuce tipburn, each with its distinct advantages and disadvantages. First, slowing down plant growth by reducing light intensity or temperature decreases calcium demand in rapidly growing tissues and thus tipburn incidence, but at the expense of yield (Ertle and Kubota, 2023b). Similarly, early harvesting before full head enclosure occurs can be a preventative measure against tipburn, but also limits yield that could otherwise be gained in the exponential growth phase. Second, foliar application of calcium chloride solutions directly supplies calcium to newer leaves and provides tipburn control (Samarakoon et al., 2020). However, frequent applications (e.g., twice a week) are needed to maintain adequate calcium levels, which increase labor costs and can lead to leaf burns or other phytotoxic effects if not applied carefully. Third, decreasing daytime relative humidity in the growing environment promotes transpiration and calcium transport (Collier and Tibbitts, 1984), but can reduce growth and is costly and difficult to regulate consistently, particularly in large-scale operations or climates with naturally high humidity. Finally, increased downward airflow from vertical airflow fans (VAFs) above the canopy promotes transpiration by dispensing humid air and decreasing the leaf boundary layer and thus limit tipburn in the enclosed lettuce head, a region often unaffected by horizontal airflow (Frantz et al., 2004). However, VAFs incur substantial capital and operating costs, block natural sunlight in greenhouses, and are challenging and costly to install in vertical farms.

Besides the above tipburn-mitigating strategies, a recent study showed that a novel chemical-based, calcium-mobilizing biostimulant completely eliminated tipburn and increased shoot fresh mass of greenhouse-grown lettuce ‘Rex’ by 28% at 21 d after transplant (DAT), and reduced tipburn by 88% without affecting shoot fresh mass at 28 DAT (Biradar and Meng, 2024). This biostimulant was administered at a relatively low concentration into the hydroponic nutrient solution at transplant and did not require replenishment. While the application of the calcium-mobilizing biostimulant had evident benefits in tipburn control, it remained unclear how it compared to and how it could be used synergistically with other tipburn-mitigating strategies such as VAFs.

The objective of this study was to investigate the combined effects of VAFs and calcium-mobilizing biostimulant application on lettuce tipburn and growth in a greenhouse environment. We hypothesized that 1) without VAFs, biostimulant application would reduce tipburn by promoting calcium mobilization to newer, rapidly growing leaves; and 2) with VAFs, the need for biostimulant application would be reduced, as improved vertical airflow and transpiration would enhance calcium uptake. By exploring the interaction between these two strategies, this study aims to offer practical solutions for optimizing lettuce quality and production efficiency for controlled-environment growers.

## Materials and methods

### Plant materials and seedling growing conditions

Green butterhead lettuce ‘Rex’ was selected for this study because it was a commercially popular cultivar with high susceptibility to tipburn despite being marketed as tolerant to tipburn. On day 0, 300 seeds (Johnny’s Selected Seeds, Winslow, ME, USA) were each sown in individual rockwool cubes (25 × 25 × 40 mm; AO 25/40, Grodan, Milton, ON, Canada). The cubes were pre-soaked with reverse-osmosis water and then placed in trays. The trays were covered with transparent humidity domes and immediately placed under fixed sole-source lighting from warm-white light-emitting diode (LED) fixtures. The daily light integral (DLI) during the germination and seedling phase was 16.2 mol·m^−2^·d^−1^ under a photoperiod of 18 h·d^−1^ (0500 to 2300 hr) and a mean photosynthetic photon flux density (PPFD; 400 to 700 nm) of 250 μmol·m^−2^·s^−1^ at the rockwool surface. The humidity domes were removed on day 3, when ≈90% of the seeds germinated. The growth room had an air temperature of at 23 ± 1°C as measured by a temperature sensor (HOBO MX1101; Onset Computer Corporation, Bourne, MA, USA) and ambient-room CO_2_.

From day 3 to day 14, seedlings were sub-irrigated with a complete nutrient solution, which was reverse-osmosis water supplemented with a base fertilizer (12N–2P–13K RO; JR Peters, Inc., Allentown, PA, USA) and magnesium sulfate (JR Peters, Inc.). The nutrient solution had an adjusted pH of 5.7 to 5.9 and an electrical conductivity (EC) of 1.2 to 1.4 dS·m^−1^ as measured using a portable meter (HI9814; Hanna Instruments, Smithfield, RI, USA). It contained the following elements (in mg·L^−1^): 125 N, 18 P, 138 K, 72 Ca, 48 Mg, 39 S, 1.8 Fe, 0.52 Mn, 0.56 Zn, 0.12 B, 0.47 Cu, and 0.13 Mo. The nutrient solution inside each tray was replenished daily to half of the rockwool cube height.

### Post-transplant greenhouse treatments

**Figure 1:**
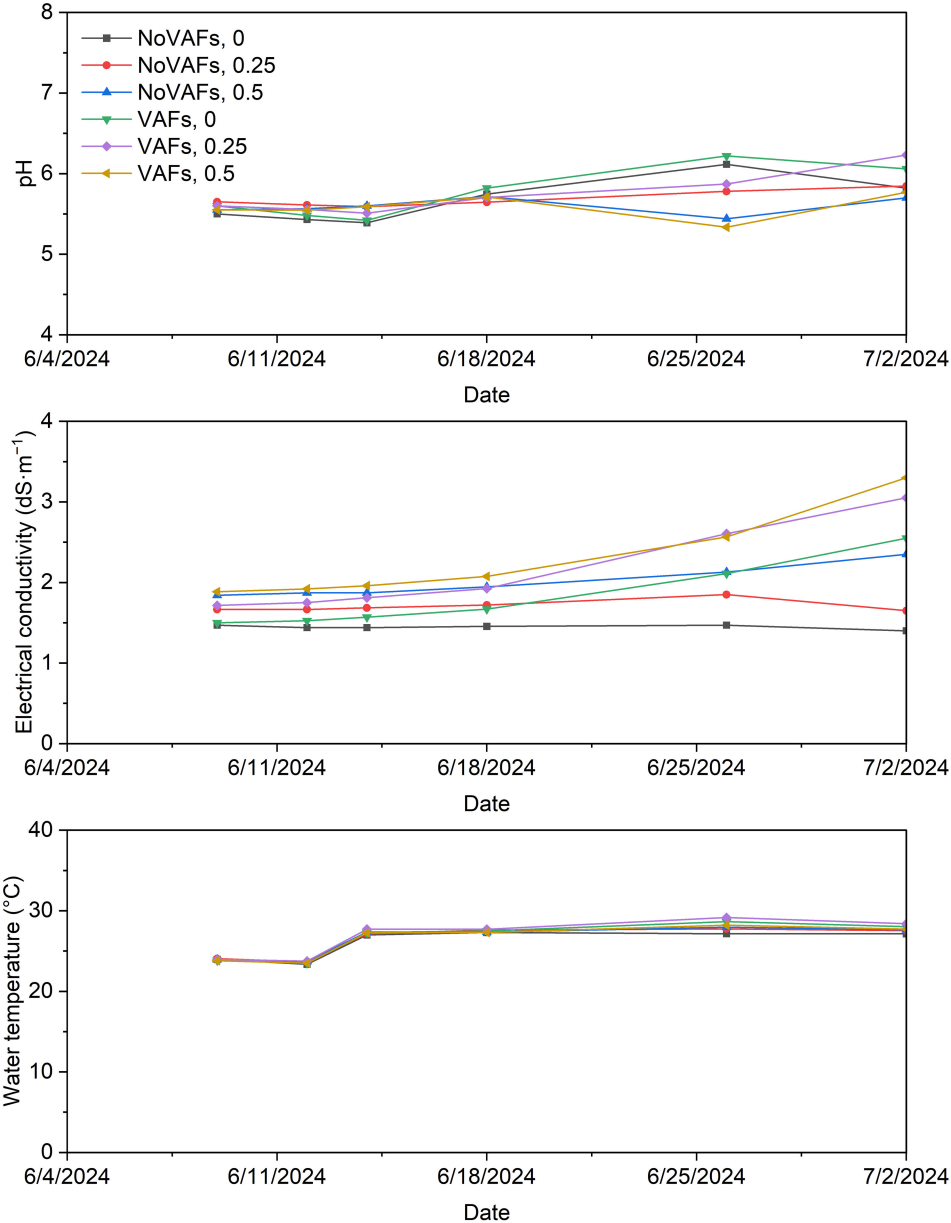
Nutrient solution pH, electrical conductivity, and temperature pooled from two blocks under all treatments throughout the experiment. Treatments consisted of a biostimulant (at concentrations of 0, 0.25, and 0.5 mL·L^−1^) with or without vertical airflow fans (VAFs).

On day 14, in a glass-glazed greenhouse (39.66° N, 75.75° W), 216 of the most uniform-looking seedlings were transplanted into 12 deep-water-culture hydroponic trays (122-cm length × 61-cm width × 20-cm depth; planting density = 24 plants·m^−2^), which were assigned to six treatments in each of two blocks in a split-plot randomized complete block design. For 28 DAT, plants were subject to three biostimulant concentrations (0, 0.25, and 0.5 mL·L^−1^, CC US-2105; Croda, Inc., Atlas Point, DE, USA) without VAFs (NoVAFs) and with VAFs. The three biostimulant concentrations were selected based on prior efficacy (~0.22 mL·L^−1^; Biradar and Meng, 2024) and preliminary screening showing no phytotoxicity at ≤0.5 mL·L^−1^ (data not shown). In each hydroponic tray, a foam raft held 18 seedlings and floated on a 12-cm-deep nutrient solution, which was made from reverse-osmosis water supplemented with 20% higher fertilizer concentrations than for seedlings to provide (in mg·L^−1^): 150 N, 22 P, 166 K, 88 Ca, 58 Mg, 47 S, 2.1 Fe, 0.63 Mn, 0.68 Zn, 0.15 B, 0.56 Cu, and 0.15 Mo. The nutrient solution pH, EC, and temperature of all treatments were measured periodically throughout the experiment with the data pooled from both blocks shown in **Figure 1**. The nutrient solution pH was adjusted to 5.7 to 5.9 three times a week with two commercial liquid buffering solutions (pH Up and pH Down; General Hydroponics, Inc., Santa Rosa, CA, USA). Initially, adding 0.25 or 0.5 mL·L^−1^ of the biostimulant increased the nutrient solution EC by ≈0.2 or 0.4 dS·m^−1^, respectively. Under NoVAFs, EC was generally steady and consistent throughout the experiment. In contrast, under VAFs, the increases in EC over time from 1.5–1.9 to 2.6–3.3 dS·m^−1^ can be attributed to increased evapotranspiration, water loss, and thus concentrated nutrients with increased air movement. Circulation pumps were not used, and the nutrient solution was not replaced or replenished during the experiment; therefore, changes in EC over time reflect gradual concentration effects and plant uptake rather than nutrient replenishment or depletion cycles. Water temperature was similar across all treatments at each time point and increased from ≈24°C to ≈28°C over time.

The biostimulant was dissolved thoroughly into the nutrient solution to supplement calcium ammonium nitrate, zinc nitrate, and ethoxylated branched C11–14, C13-rich alcohols (chemical structures and percentages are proprietary). No specific devices were used for the purpose of aeration but a small submersible water pump (5W, 95 GPH, model PL-128-1; Pulaco, Guangzhou, Guangdong Province, China) was installed in each tray to circulate the nutrient solution during the whole experiment, which also mixed air in water via bubbling on the surface. No further water, fertilizers, or biostimulant were added after transplant. The nutrient solution was not refreshed, changed, or maintained at a specific level because the hydroponic trays held enough nutrient solutions to sustain the plants throughout the entire production phase.

Four medium-sized fans (30 cm in diameter, 60 m^3^·min^−1^ of airflow; Simple Deluxe, Duarte, CA, USA) were installed 120 cm above the plants that received VAFs to provide a consistent air velocity of 1.0 ± 0.2 m·s^−1^ without affecting light uniformity. Air velocity was measured at planting sites within each VAF treatment area using an anemometer (Anemomaster Lite, Model 6006-DE; Kanomax USA, Inc., Andover, NJ, USA) equipped with a single-direction sensor. The fans were connected to a timer and operated for 12 h·d^−1^ from 0600 to 1800 hr. The fans did not cause shading that consistently affected the DLI.

### Greenhouse environmental conditions

**Figure 2:**
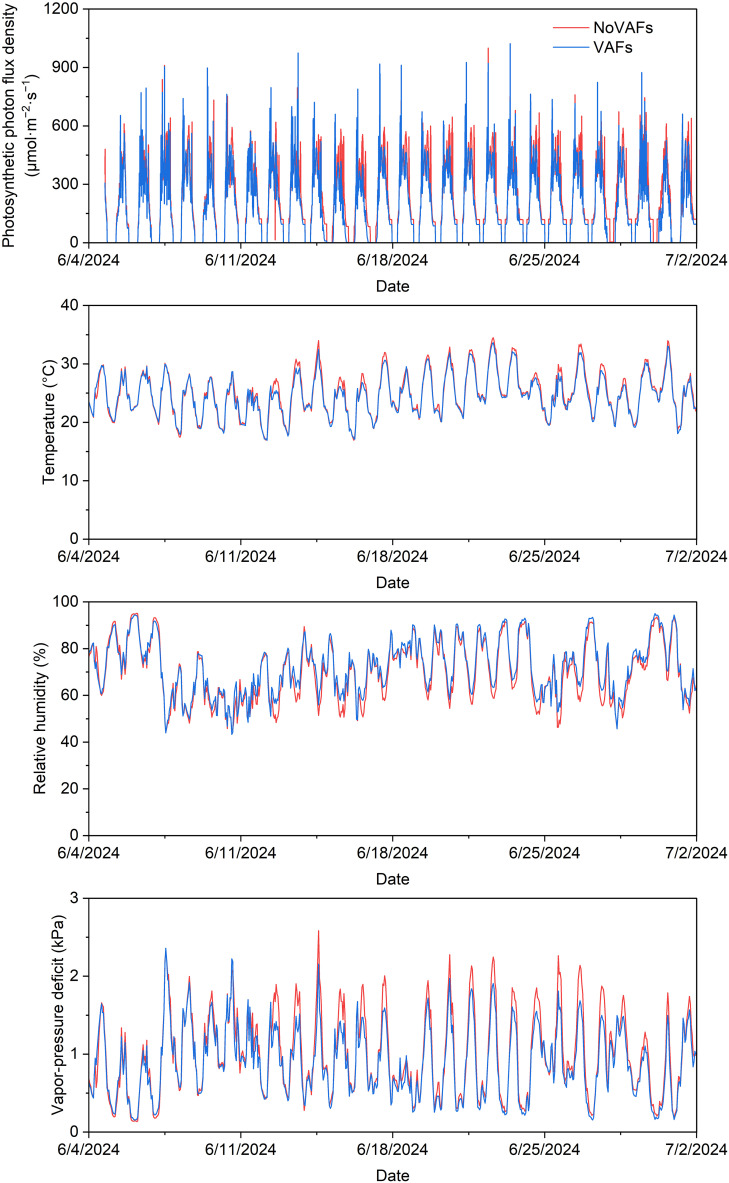
Photosynthetic photon flux density, air temperature, relative humidity, and vapor-pressure deficit recorded on an hourly basis throughout the greenhouse experiment in treatment areas with and without vertical airflow fans (VAFs).

Plants were grown under sunlight and supplemental lighting from cool-white + red LED fixtures (VL1; Volt Grow, Lutz, FL, USA), which helped to achieve the target daily light integral (DLI) and maintain an 18-h photoperiod (0500–2300 hr). The greenhouse had shade screens to reduce heat from solar radiation and a fan-and-pad cooling system to decrease air temperature during summer months of June and July, when the experiment was conducted. Four temperature and relative humidity sensors (HOBO MX2301; Onset Computer Corporation, Bourne, MA, USA) were placed across the experimental area to log data every 10 min. Additionally, four full-spectrum quantum sensors (SQ-500; Apogee Instruments, Inc., Logan, UT, USA) were used to log the PPFD every 30 min. The mean photosynthetic photon flux density (over 24 h) and the mean DLI across all treatments throughout the experiment were 200.3 μmol·m^−2^·s^−1^ and 17.3 mol·m^−2^·d^−1^, respectively. The air temperature, relative humidity, and vapor-pressure deficit (mean ± *SD*) throughout the experiment were 24.8 ± 3.7°C, 70.3% ± 12.2%, and 0.98 ± 0.52 kPa, respectively, without VAFs, and 24.4 ± 3.5°C, 72.4% ± 11.5%, and 0.88 ± 0.45 kPa, respectively, with VAFs. Hourly data for these environmental parameters without and with VAFs are shown in **Figure 2**.

### Data collection and analysis

**Figure 3:**
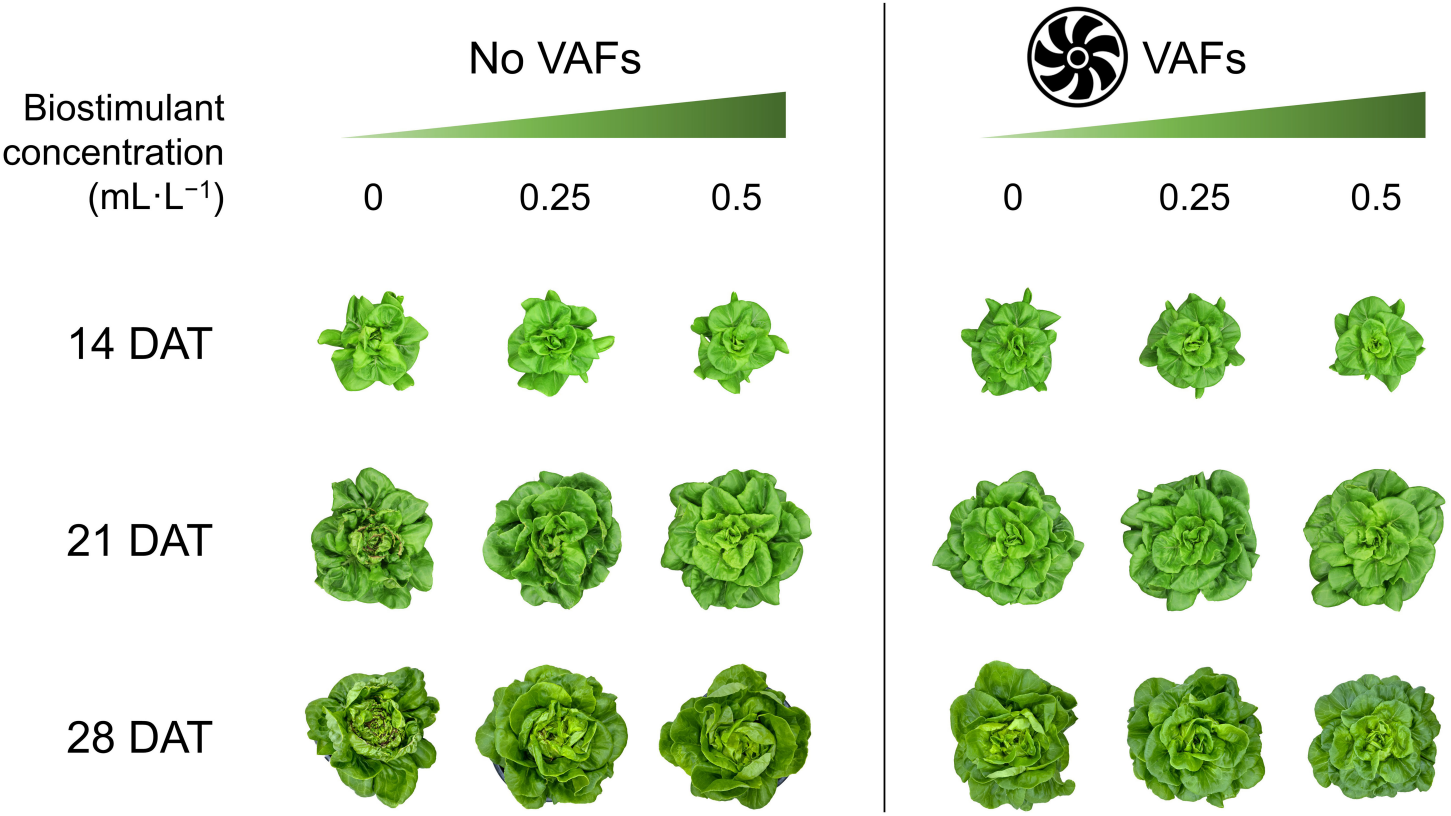
Representative plant images of hydroponically grown lettuce ‘Rex’ treated with a biostimulant (at concentrations of 0, 0.25, and 0.5 mL·L^−1^) with or without vertical airflow fans (VAFs) at 14, 21, and 28 d after transplant (DAT).

**Figure 4:**
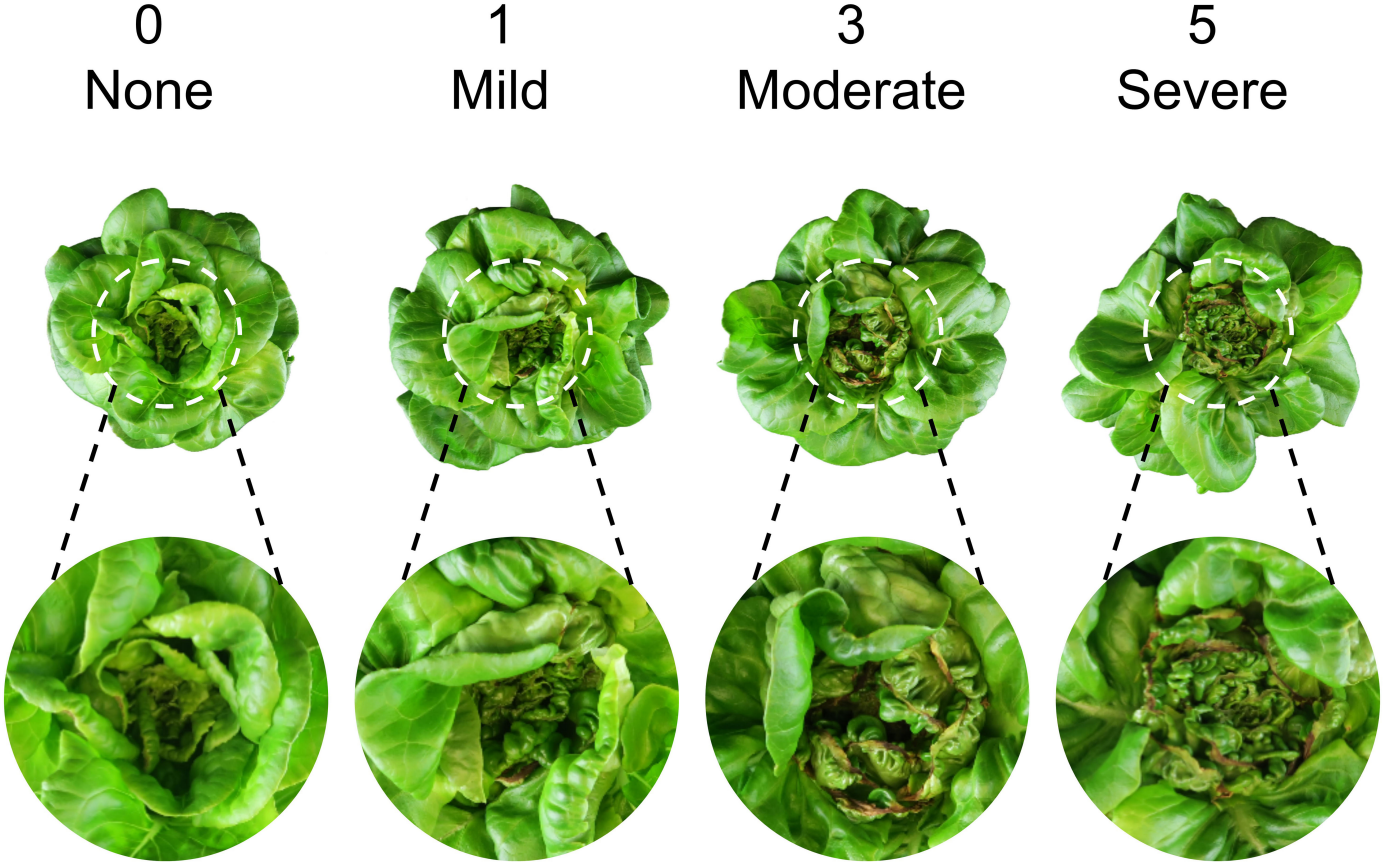
Reference images illustrating lettuce ‘Rex’ tipburn severity ratings used for phenotyping (0 = none, 1 = mild, 3 = moderate, 5 = severe).

Each treatment combination was replicated in two blocks, with one tray per treatment per block serving as the experimental unit. Plants were sampled from all 12 hydroponic trays (experimental units) at 14, 21, and 28 DAT early in the morning. Before each data collection, a representative plant per tray was photographed to capture plant appearance under treatments (**Figure 3**). To minimize temporal variations, a total of 48 plants were sampled simultaneously from all treatments at each harvest time (i.e., four plants as subsamples per treatment per block, and eight plants as subsamples per treatment across both blocks). After separating shoots from roots, we measured shoot fresh mass and photographed one representative plant per treatment per block from the top view. The plant diameter (i.e., the average of two horizontal distances between outer leaf edges) was measured using a ruler. The crown diameter (i.e., the thickness of the connecting part between roots and shoot) was also measured by a caliper. The tipburn severity of each plant was visually rated on a 0–5 scale of (0 = none, 1 = mild, 3 = moderate, 5 = severe), following the protocols of Biradar and Meng (2024). Representative close-up images of tipburn severity corresponding to each rating anchor (0, 1, 3, and 5) are shown in **Figure 4**. The same rater conducted all evaluations independently to ensure consistency and reproducibility. Leaves with and without tipburn were counted separately. The ratio of the number of leaves (≥2 cm in length) with tipburn to the total number of leaves per plant (percentage of leaves with tipburn) was calculated as an additional measure of tipburn severity. The chlorophyll concentration index was measured at three random positions on a mature, fully expanded leaf of each plant using a handheld chlorophyll meter (MC-100, Apogee Instruments). Samples were labeled, bagged, and placed in a forced-air drying oven (SMO28–2; Sheldon Manufacturing, Inc., Cornelius, OR, USA) at 60°C for a minimum of 72 h. Dried leaf tissue (≈20 leaves) from the center of each plant were taken for subsequent nutrient analysis only for the last harvest (28 DAT). Nutrient analysis of plant inner tissue samples was performed at the University of Delaware Soil Testing Laboratory (Newark, DE, USA). Moisture content was calculated as the percentage of water relative to shoot fresh mass [(shoot fresh mass – dry mass) ÷ shoot fresh mass × 100].

Data were analyzed separately for each harvest date (14, 21, and 28 DAT) in statistical software JMP (JMP Student Edition; SAS Institute Inc., Cary, NC, USA). The experiment was a split-plot randomized complete block design, with VAFs as the whole-plot factor, biostimulant concentration as the split-plot factor, and two blocks. Within each block and treatment combination, four plants were sampled as subsamples. Mixed-effects models were used to account for the split-plot structure, with VAFs, biostimulant concentration, and their interaction treated as fixed effects and block as a random effect. The whole-plot error term (used to test the effect of VAFs) was estimated from the variability among whole plots within blocks, while the split-plot error term (used to test the effect of biostimulant and the VAFs × biostimulant interaction) was estimated from the variability among split plots within each whole plot. Residuals were inspected and passed checks for normality and homoscedasticity. All parameters were analyzed using analysis of variance followed by Tukey’s honest significant difference test (*α* = 0.05) of the VAFs × biostimulant factor to compare treatment means. Exact *P*-values for main and interaction effects on all parameters and dates are reported in **Table 1**. Data visualization for all parameters and linear regression analysis for relationships between inner tissue calcium concentration and the tipburn rating or the percentage of leaves with tipburn were performed in the Origin software (Origin 2023; OriginLab Corporation, Northampton, MA, USA).

**Table 1:** Exact *P*-values for fixed effects [vertical airflow fans (VAFs), biostimulant, and VAFs*Biostimulant] on all plant parameters and days after transplant (DAT).

Parameter	Tipburn rating			Percentage of leaves with tipburn		
DAT	14	21	28	14	21	28
VAFs	0.0004	<0.0001	<0.0001	0.0114	<0.0001	<0.0001
Biostimulant	<0.0001	<0.0001	<0.0001	0.0024	<0.0001	0.0103
VAFs*Biostimulant	<0.0001	<0.0001	<0.0001	0.0024	<0.0001	0.0168

Parameter	Total leaf number			Shoot fresh mass		
DAT	14	21	28	14	21	28
VAFs	0.1350	0.1134	0.0060	0.3131	0.0048	0.0942
Biostimulant	0.1734	0.5168	0.2020	<0.0001	0.0003	0.0037
VAFs*Biostimulant	0.7713	0.0379	0.4394	0.3205	0.1876	0.0397

Parameter	Shoot dry mass			Moisture content		
DAT	14	21	28	14	21	28
VAFs	0.1760	0.0073	0.4874	0.5243	0.0102	0.0774
Biostimulant	<0.0001	0.0838	0.0330	<0.0001	<0.0001	<0.0001
VAFs*Biostimulant	0.3099	0.4382	0.1995	0.9983	0.0431	0.0878

Parameter	Plant diameter			Crown diameter		
DAT	14	21	28	14	21	28
VAFs	No data	0.4407	0.0005	0.6470	0.0621	0.2546
Biostimulant	No data	0.0120	<0.0001	0.0003	0.0034	0.1941
VAFs*Biostimulant	No data	0.4766	<0.0001	0.5951	0.3855	0.4605

Parameter	Chlorophyll concentration index			Inner tissue calcium concentration		
DAT	14	21	28	14	21	28
VAFs	0.0626	0.0020	0.5852	No data	No data	<0.0001
Biostimulant	0.0042	0.5812	0.0332	No data	No data	<0.0001
VAFs*Biostimulant	0.1650	0.5671	0.0044	No data	No data	0.0811

## Results

### Tipburn rating, percentage of leaves with tipburn, and total leaf number

**Figure 5:**
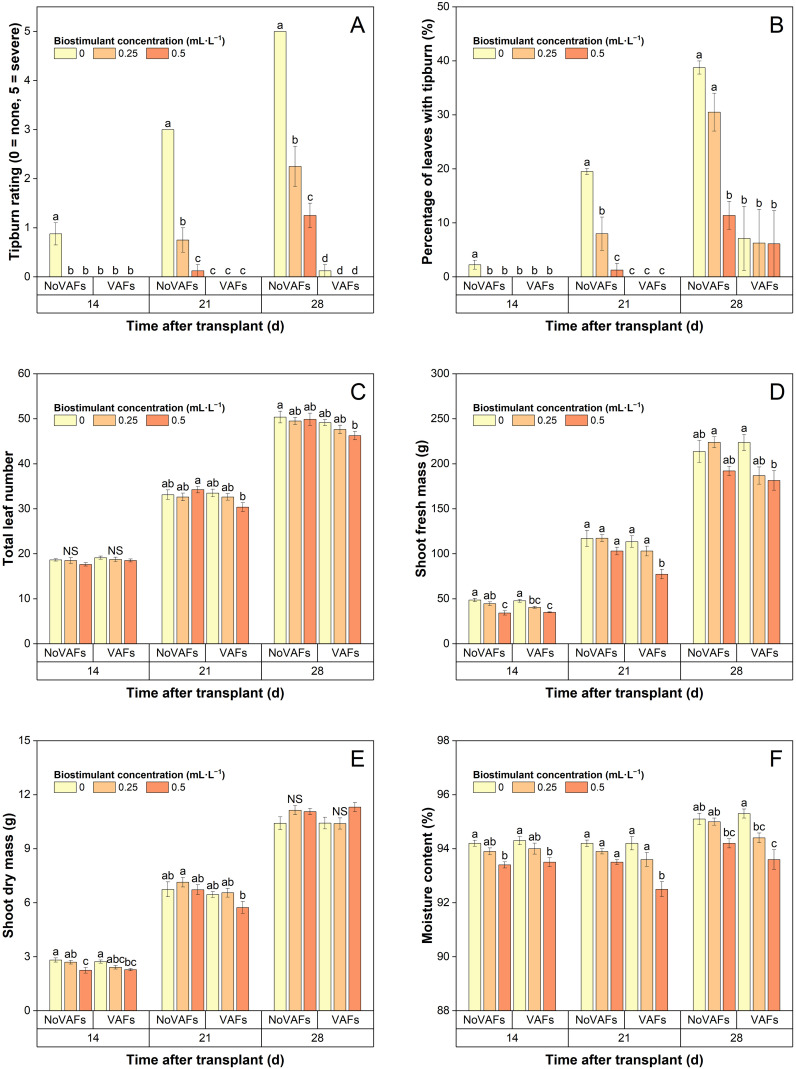
Tipburn rating **(A)**, percentage of leaves with tipburn **(B)**, total leaf number **(C)**, shoot fresh mass **(D)**, shoot dry mass **(E)**, and moisture content **(F)** of hydroponically grown lettuce ‘Rex’ treated with a biostimulant (at concentrations of 0, 0.25, and 0.5 mL·L^−1^) with or without vertical airflow fans (VAFs). Different letters within each harvest time indicate significant differences (Tukey’s honest significant difference test, *α* = 0.05). NS, non-significant. Values are means ± *SE*. At each time point, data from each treatment combination in each of two blocks were taken from four plant subsamples.

The tipburn rating (and the percentage of leaves with tipburn) in plants with NoVAFs and no biostimulant (control) increased from 0.9 (2% burnt leaves) at 14 DAT to 3.0 (20% burnt leaves) at 21 DAT and to 5.0 (39% burnt leaves) at 28 DAT (**Figures 3**, **5A**, **5B**). At 14 DAT, no tipburn was present in any treatments except the control group. At 21 DAT, tipburn was present in lettuce grown under NoVAFs, but not under VAFs. Increasing the biostimulant concentration from 0 to 0.5 mL·L^−1^ decreased the tipburn rating from 3.0 to 0.1 and the percentage of burnt leaves from 20% to 1%. At 28 DAT, tipburn worsened in lettuce grown under NoVAFs, but was minimal under VAFs (≤0.1 tipburn rating; ≤7% burnt leaves in the central enclosed head). Increasing the biostimulant concentration from 0 to 0.5 mL·L^−1^ decreased the tipburn rating from 5.0 to 1.3 and the percentage of burnt leaves from 39% to 11%. At 14 DAT, total leaf number was similar across all six treatments (**Figure 5C**). At 21 DAT, compared to the control, total leaf number all the other treatments was similar. At the highest biostimulant concentration (0.5 mL·L^−1^), plants had 4 more (13% more) leaves under NoVAFs than under VAFs. At 28 DAT, plants had 4 fewer (8% fewer) leaves at the highest biostimulant concentration under VAFs compared to the control.

### Shoot fresh mass, shoot dry mass, and moisture content

At 14 DAT, increasing the biostimulant concentration from 0 to 0.5 mL·L^−1^ decreased shoot fresh mass by 26%–30% regardless of VAFs, while VAFs did not influence shoot fresh mass regardless of biostimulant concentrations (**Figure 5D**). At 21 DAT, increasing the biostimulant concentration from 0 to 0.5 mL·L^−1^ did not affect shoot fresh mass under NoVAFs but decreased it by 32% under VAFs. Shoot fresh mass was unaffected by VAFs except that it was 25% lower under VAFs than under NoVAFs at the highest biostimulant concentration (0.5 mL·L^−1^). At 28 DAT, compared to the control group, shoot fresh mass under all the other treatments was comparable. Increasing the biostimulant concentration from 0 to 0.5 mL·L^−1^ did not affect shoot fresh mass under NoVAFs but decreased it by 19% under VAFs. Shoot fresh mass was 17% lower under VAFs than under NoVAFs at the intermediate biostimulant concentration (0.25 mL·L^−1^).

At 14 DAT, increasing the biostimulant concentration from 0 to 0.5 mL·L^−1^ decreased shoot dry mass by 16%–20% regardless of VAFs, but this difference was no longer evident at 21 or 28 DAT (**Figure 5E**). Shoot dry mass was unaffected by VAFs regardless of biostimulant concentrations on all harvest dates. Increasing the biostimulant concentration from 0 to 0.5 mL·L^−1^ decreased moisture content by 0.8% regardless of VAFs at 14 DAT and by 1.8% under VAFs, but not under NoVAFs, at 21 and 28 DAT (**Figure 5F**). Moisture content was unaffected by VAFs except that it was 1.1% lower under VAFs than under NoVAFs at the highest biostimulant concentration (0.5 mL·L^−1^).

### Plant diameter and crown diameter

**Figure 6:**
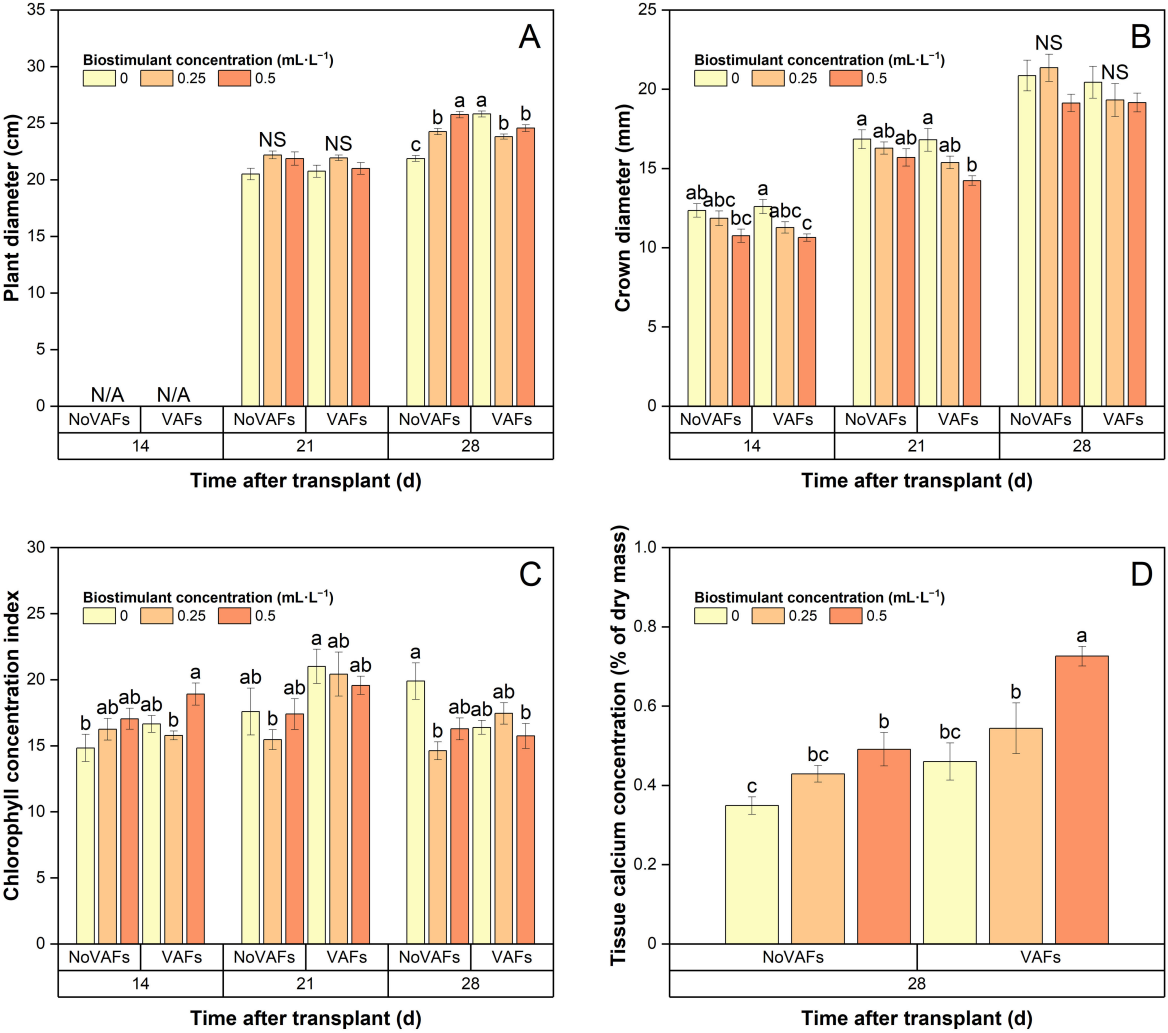
Plant diameter **(A)**, crown diameter **(B)**, chlorophyll concentration index **(C)**, and inner tissue calcium concentration **(D)** of hydroponically grown lettuce ‘Rex’ treated with a biostimulant (at concentrations of 0, 0.25, and 0.5 mL·L^−1^) with or without vertical airflow fans (VAFs). Different letters within each harvest time indicate significant differences (Tukey’s honest significant difference test, *α* = 0.05). N/A, not applicable (data not collected). NS, non-significant. Values are means ± *SE*. At each time point, data from each treatment combination in each of two blocks were taken from four plant subsamples.

Plant diameter was not measured at 14 DAT and was similar across all treatments at 21 DAT (**Figure 6A**). At 28 DAT, plant diameter was the smallest in the control group and increased or decreased with increasing biostimulant concentrations under NoVAFs or VAFs, respectively. Compared to NoVAFs, VAFs increased plant diameter by 18% with no biostimulant, did not affect it at the intermediate biostimulant concentration (0.25 mL·L^−1^), and decreased it by 5% at the highest biostimulant concentration (0.5 mL·L^−1^).

At 14 and 21 DAT, increasing the biostimulant concentration from 0 to 0.5 mL·L^−1^ decreased crown diameter by 15% under VAFs, but not under NoVAFs (**Figure 6B**). At 28 DAT, crown diameter was not affected by the biostimulant application. At all times, VAFs did not affect crown diameter regardless of biostimulant concentrations.

### Chlorophyll concentration index and plant inner tissue calcium concentration

At all times, the biostimulant concentration or VAFs generally did not affect chlorophyll concentration index with the following exceptions (**Figure 6C**). At 14 DAT, compared to the control group, plants with VAFs and 0.5 mL·L^−1^ biostimulant had 28% higher chlorophyll concentration index. At 21 DAT, chlorophyll concentration index was 35% higher under VAFs and no biostimulant than under NoVAFs and 0.25 mL·L^−1^ biostimulant. At 28 DAT, compared to the control group, chlorophyll concentration index was 21%–27% lower under NoVAFs and0.25 mL·L^−1^ biostimulant or under VAFs and 0.5 mL·L^−1^ biostimulant.

At 28 DAT, inner tissue calcium concentration was the lowest in plants grown under NoVAFs and no biostimulant (**Figure 6D**). Increasing the biostimulant concentration from 0 to 0.5 mL·L^−1^ increased inner tissue calcium concentration by 41% and 58% under NoVAFs and VAFs, respectively. Compared to NoVAFs, VAFs increased inner tissue calcium concentration by 48% at the highest biostimulant concentration (0.5 mL·L^−1^).

## Discussion

Compared to the control, the calcium-mobilizing chemical biostimulant at a concentration of 0.25 or 0.5 mL·L^−1^ showed high efficacy at controlling tipburn in greenhouse hydroponic lettuce ‘Rex’ (by decreasing the tipburn rating and the percentage of leaves with tipburn by up to 71%–75%) without influencing biomass under minimal air movement. There was a dose-dependent response, in which the biostimulant efficacy increased with increasing concentrations up to 0.5 mL·L^−1^. Similarly, in a previous greenhouse study, this biostimulant at a concentration of 0.22 mL·L^−1^ decreased the tipburn rating and number of leaves with tipburn in lettuce ‘Rex’ by up to 85%–88% compared with no biostimulant (Biradar and Meng, 2024). Both studies were conducted in summer for the same duration (up to 28 DAT) under similar air temperature (24–25 °C) and relative humidity (74%–77%), which were conducive to fast plant growth and low transpiration. Although the DLI in this study (17.3 mol·m^−2^·d^−1^) was 23% lower than that in the previous study (22.5 mol·m^−2^·d^−1^), it was sufficiently high to achieve high lettuce growth rates and induce tipburn under minimal air movement (e.g., <0.2 m·s^−1^) (Ertle and Kubota, 2023a; Ertle and Kubota, 2023b). Under NoVAFs, the biostimulant reduced tipburn compared to the control without affecting shoot fresh mass, while increasing plant diameter. This apparent increase in diameter likely reflects improved lettuce head expansion, as tipburn-affected plants developed curled inner leaves that limited expansion and reduced the overall canopy size.

Although growing conditions and methods were relatively similar, a higher biostimulant concentration in this study (0.5 mL·L^−1^) was required to elicit similar tipburn control compared to the lower biostimulant concentration in the previous study (0.22 mL·L^−1^) (Biradar and Meng, 2024). This could be at least partly attributed to varying greenhouse air dynamics potentially causing differences in plant transpiration. Side exhaust fans were used for cooling and ventilation in both studies, but they were ≥2.5 m and ≥0.8 m away from the hydroponic trays in this study and the previous study, respectively. In addition, overhead horizontal airflow fans were used constantly in the previous study, but not this study. Promotion of horizontal airflow can reduce tipburn occurrence and severity in lettuce, although not as effective as VAFs (Ahmed et al., 2020; Kaufmann, 2023; Lee et al., 2013). Compared with this study, the previous study likely had greater air movement at the plant level to facilitate plant transpiration and calcium transport and thus mitigate tipburn to a certain extent, thereby lowering the required biostimulant concentration for enhanced calcium mobility and tipburn control. Concentrations above 0.5 mL·L^−1^ were not tested in this study because preliminary trials indicated potential phytotoxic effects, and our focus was to evaluate the interaction of VAFs and the biostimulant within a safe, agronomically relevant range.

Compared to the control, VAFs consistently minimized tipburn (by up to 100%) in this study under a DLI of 17.3 mol·m^−2^·d^−1^. Similarly, VAFs eliminated tipburn in greenhouse hydroponic lettuce ‘Ostinata’ under a DLI of ≤17 mol·m^−2^·d^−1^, whereas tipburn developed despite VAFs under a higher DLI of 20 or 22 mol·m^−2^·d^−1^ (Both et al., 1997). Moreover, supply of air with a high vapor-pressure deficit (0.74 kPa at 25 °C temperature and 76% relative humidity) to inner lettuce leaves increased transpiration and thus water and calcium uptake from the roots as well as calcium distribution to the inner leaves (increased by 3.6 times compared to the control) (Goto and Takakura, 1992a). The vapor-pressure deficit, temperature, and relative humidity of the ambient air in this study were similar with or without VAFs (on average 0.88–0.98 kPa at 24–25°C temperature and 70%–73% relative humidity, **Figure 2**), indicating that differences in ambient climate were not the primary driver of treatment effects. The vapor-pressure deficit fluctuated throughout the day, following temperature fluctuations, with higher daytime values (often in the 1.0–2.5 kPa range) (**Figure 2**). Therefore, the vapor-pressure deficit of the air blown downwards to the plant centers by VAFs was sufficiently high to increase transpiration and calcium delivery to the shoot tip. In addition, increased air movement from VAFs can increase boundary layer conductance and thus transpiration and mass-flow-driven calcium mobilization (Ahmed et al., 2022). The VAFs in this study ran for 12 h·d^−1^ (0600 to 1800 hr) under an 18-h photoperiod (0500 to 2300 hr) to increase transpiration when stomata were open, which is consistent with recommendations to promote airflow during daytime or continuously for tipburn control (Goto and Takakura, 1992a).

As the ambient vapor-pressure deficit increased, calcium accumulation in lettuce without air supply increased linearly in outer leaves but remained low in inner leaves (Goto and Takakura, 1992a). A wide range of head and leaf lettuce types form tightly enclosed leaves at the shoot tip, trapping humid air inside regardless of outside humidity. This suggests that decreasing relative humidity at a given temperature to increase the ambient vapor-pressure deficit cannot promote calcium delivery to the shoot tip to alleviate tipburn. Both the biostimulant and VAFs bypass this barrier and improve calcium distribution to inner leaves via the symplastic pathway and the apoplastic pathway, respectively (Biradar and Meng, 2024). The biostimulant contains calcium ammonium nitrate, zinc nitrate, and ethoxylated branched alcohols, which has been suggested to stimulate selective membrane ion transport and thereby enhance local calcium mobility when apoplastic flow is limited (Croda, 2020, manufacturer technical communication). This plausibly aligns with the dose-responsive reductions in tipburn we observed under NoVAFs. However, our study did not directly assess calcium speciation, xylem loading, or apoplastic versus symplastic partitioning. Targeted ionomic and tracer-based approaches will be needed to validate the mechanistic basis of the biostimulant-driven calcium redistribution.

**Figure 7:**
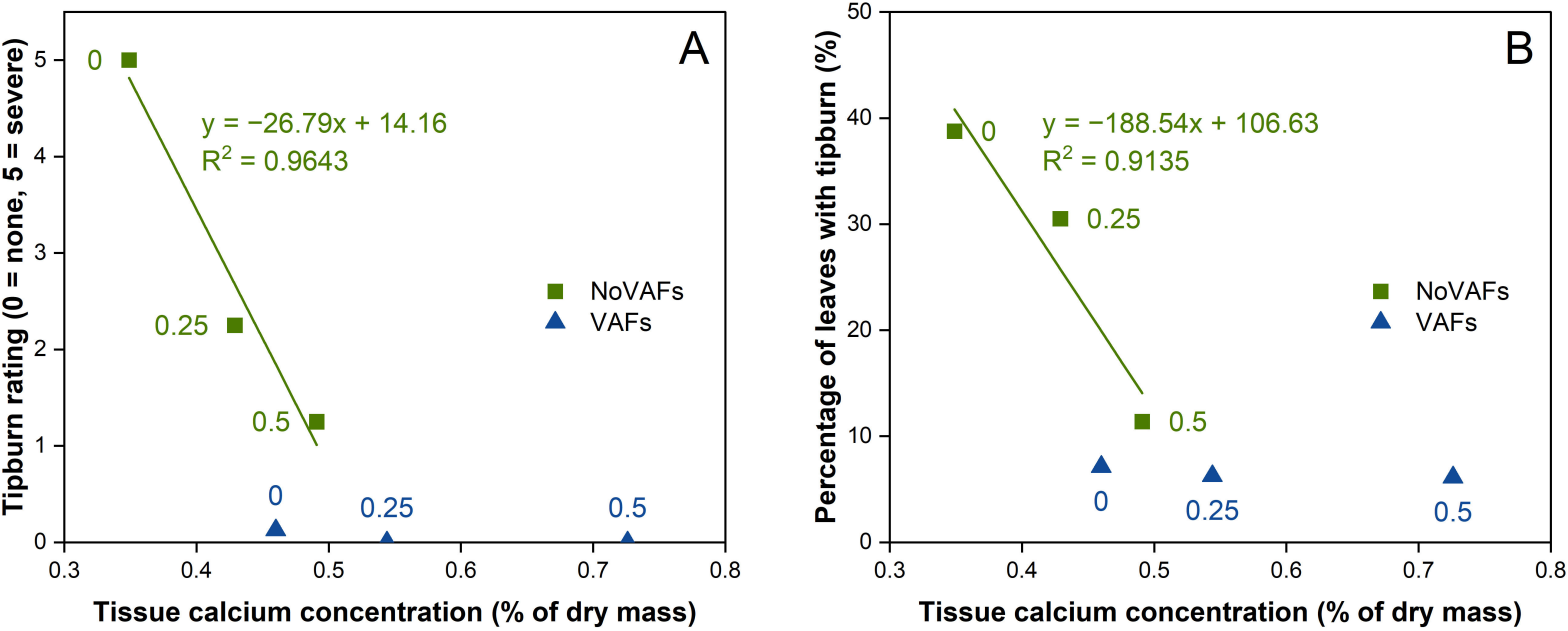
Relationships between inner tissue calcium concentration and tipburn rating **(A)** or percentage of leaves with tipburn **(B)** of hydroponically grown lettuce ‘Rex’ treated with a biostimulant (at concentrations of 0, 0.25, and 0.5 mL·L^−1^) with or without vertical airflow fans (VAFs). Linear regression equations and R^2^ values are shown for data under NoVAFs.

In this study, we quantified calcium concentration in inner leaf tissues at 28 DAT to assess whether treatment effects on tipburn mitigation were associated with improved calcium delivery to the shoot tip. The biostimulant at a concentration of 0.5 mL·L^−1^ increased lettuce inner tissue calcium concentration compared to no biostimulant under no VAFs. There was an inverse linear relationship between inner tissue calcium concentration and tipburn severity, whether quantified as the tipburn rating (**Figure 7A**, tipburn rating = −26.79 × [Ca] + 14.16, R^2^ = 0.9643) or the percentage of leaves with tipburn (**Figure 7B**, percentage of leaves with tipburn = −188.54 × [Ca] + 106.63, R^2^ = 0.9135). In contrast, there was no such correlation under VAFs, where inner tissue calcium concentration was similarly sufficient irrespective of the biostimulant. Our data revealed an interaction between the biostimulant and VAFs. When VAFs were not used, the biostimulant at a sufficient concentration (0.2–0.5 mL·L^−1^) was generally comparable to VAFs at controlling tipburn. When used alone, VAFs were highly effective, thereby rendering no need for further promotion in calcium delivery through the biostimulant. The within-cultivar correlations between inner-leaf calcium and tipburn severity are consistent with genotype-dependent nutrient patterns reported by Birlanga et al. (2021), highlighting that both airflow-mediated boundary layer effects and chemically enhanced calcium mobility may vary across lettuce genotypes and motivating future studies of treatment × genotype interactions. Because tissue calcium concentration was measured only at 28 DAT, interpretations regarding earlier stages are limited to observed phenotypic trends.

With a few exceptions, VAFs in this study generally did not influence lettuce leaf number, biomass (decreased only in a few instances), plant size, or chlorophyll concentration index. Similarly, VAFs did not influence or slightly decreased shoot fresh mass in lettuce ‘Klee’ and ‘Rouxai’ grown indoors under a DLI of 15 mol·m^−2^·d^−1^ (Kaufmann, 2023). Moderate airflow can potentially decrease plant growth when mechanical stimulation elicits the thigmomorphogenic signaling pathway, which limits extension growth and diverts resources to structural reinforcement (Grace, 1988). In this study, no visible canopy deformation or excessive leaf flutter was observed under the applied airflow levels, suggesting that thigmomorphogenic growth inhibition was minimal. In addition, VAFs did not negatively influence biomass production in lettuce ‘Ostinata’ (Both et al., 1997). While VAFs generally do not increase shoot fresh mass, targeted air supply to the shoot tip can increase shoot fresh mass. For example, targeted meristem aeration with air tubes minimized tipburn while increasing shoot fresh mass per unit area in mature lettuce ‘Waldmann’s Green’ and ‘Buttercrunch’ (Frantz et al., 2004). In addition, supplying targeted air to inner lettuce leaves through air tubes slightly increased shoot fresh mass but did not influence water content or chlorophyll content (Goto and Takakura, 1992b).

The transient decreases in shoot fresh mass under increasing biostimulant concentrations at early time points (14 and 21 DAT) were associated with reductions in both shoot dry mass and tissue moisture content. By 28 DAT, shoot dry mass was unaffected by either VAFs or biostimulant concentration, indicating comparable biomass accumulation among treatments. However, under VAFs, increasing the biostimulant concentration reduced moisture content slightly (by 1.8%), which was sufficient to lower shoot fresh mass by 19% despite unchanged dry mass. Because lettuce tissues contain more than 90% water, even small changes in moisture fraction can substantially influence fresh mass. The lower moisture content likely reflects slightly greater transpiration and mild tissue dehydration under airflow, potentially reinforced by enhanced calcium-mediated cell wall rigidity, rather than a reduction in actual growth. Overall, these transient or minor fresh-mass reductions did not persist to final harvest under no VAFs and therefore do not represent a yield penalty.

In conclusion, the calcium-mobilizing biostimulant tested in this study provided comparable tipburn mitigation in hydroponic lettuce to VAFs under summer greenhouse conditions where VAFs may be impractical, without causing a yield penalty at final harvest. Although minor or transient reductions in shoot fresh mass occurred at early harvests due to temporary decreases in dry mass and tissue moisture, these effects did not persist by 28 DAT. The biostimulant improved inner-leaf calcium levels under no VAFs, correlating with reduced tipburn severity, while VAFs effectively promoted calcium delivery through enhanced air movement. Together, these findings support the use of the biostimulant as a practical alternative or complement to mechanical airflow for tipburn control in hydroponic lettuce. Under different DLI, temperature, VPD, or airflow regimes, combined strategies or different doses may be necessary.

## Data Availability

The raw data supporting the conclusions of this article will be made available by the authors, without undue reservation.
